# The Demographic History of Populations and Genomic Imprinting have Shaped the Transposon Patterns in *Arabidopsis lyrata*

**DOI:** 10.1093/molbev/msaf093

**Published:** 2025-04-24

**Authors:** Nélida Padilla-García, Audrey Le Veve, Vojtěch Čermák, Ömer İltaş, Adrián Contreras-Garrido, Sylvain Legrand, Jean-Marc Aury, Robert Horvath, Clément Lafon Placette

**Affiliations:** Department of Botany, Faculty of Science, Charles University, Prague, Czech Republic; Departamento de Botánica y Fisiología Vegetal, Universidad de Salamanca, 37007 Salamanca, Spain; Department of Botany, Faculty of Science, Charles University, Prague, Czech Republic; Department of Botany, Faculty of Science, Charles University, Prague, Czech Republic; Department of Experimental Plant Biology, Faculty of Science, Charles University, Prague, Czech Republic; Department of Botany, Faculty of Science, Charles University, Prague, Czech Republic; Univ. Lille, CNRS, UMR 8198—Evo-Eco-Paleo, F-59000 Lille, France; Univ. Lille, CNRS, UMR 8198—Evo-Eco-Paleo, F-59000 Lille, France; Génomique Métabolique, Genoscope, Institut François Jacob, CEA, CNRS, Univ Evry, Université Paris—Saclay, 91057 Evry, France; Forest Genetics, Albert-Ludwigs-Universität Freiburg, Bertoldstr. 17, Freiburg, Germany; Department of Plant and Microbial Biology, University of Zurich, Zurich, Switzerland; Department of Botany, Faculty of Science, Charles University, Prague, Czech Republic

**Keywords:** transposable elements, *Arabidopsis lyrata*, transposon load, demographic history, genomic imprinting, positive selection

## Abstract

Purifying selection is expected to prevent the accumulation of transposable elements (TEs) within their host, especially when located in and around genes and if affected by epigenetic silencing. However, positive selection may favor the spread of TEs, causing genomic imprinting under parental conflict, as genomic imprinting allows parent-specific influence over resource accumulation to the progeny. Concomitantly, the number and frequency of TE insertions in natural populations are conditioned by demographic events. In this study, we aimed to test how demography and selective forces interact to affect the accumulation of TEs around genes, depending on their epigenetic silencing, with a particular focus on imprinted genes. To this aim, we compared the frequency and distribution of TEs in *Arabidopsis lyrata* from Europe and North America. Generally, we found that TE insertions showed a lower frequency when they were inserted in or near genes, especially TEs targeted by epigenetic silencing, suggesting purifying selection at work. We also found that many TEs were lost or got fixed in North American populations during the colonization and the postglacial range expansion from refugia of the species in North America, as well as during the transition to selfing, suggesting a potential “TE load.” Finally, we found that silenced TEs increased in frequency and even tended to reach fixation when they were linked to imprinted genes. We conclude that in *A. lyrata*, genomic imprinting has spread in natural populations through demographic events and positive selection acting on silenced TEs, potentially under a parental conflict scenario.

## Introduction

Genomic imprinting is an epigenetic phenomenon that leads to gene expression in a parent-of-origin specific manner (Batista and Köhler 2020). It is one of the rare cases of convergent evolution between angiosperms and placental mammals. In both cases, genomic imprinting has evolved independently in a biparental structure that nourishes the embryo—the endosperm for angiosperms and the placenta for mammals (Pires and Grossniklaus 2014). Also, both clades are viviparous. The convergent evolution of genomic imprinting in mammals and angiosperms is thought to be a consequence of these similarities, which altogether set the stage for parental conflict over resource allocation to the developing embryo. According to the parental conflict or kinship theory, the interest of paternally derived genes in plants would be to promote the accumulation of resources in the endosperm to maximize their availability for their offspring and thus, their chances to survive. In contrast, the effect of the maternally derived genes would be to limit resource allocation in the endosperm, as the maternal interest is to balance the resources to all her potential future offspring (Geist et al. 2019). In this context, genomic imprinting would have evolved as a molecular trait allowing antagonistic and parent-specific influence on this allocation (Pires and Grossniklaus 2014). This conflict is expected to be stronger in outcrossing than in selfing plants because in selfers, the same individual provides both gametes to the seeds (Petrén et al. 2023). One manifestation of this difference is the “weak inbreeder strong outbreeder” phenomenon (Brandvain and Haig 2005): outcrossers tend to show a stronger parent-specific influence on endosperm development than selfers, with imprinted genes suggested as underlying candidates (Lafon-Placette and Köhler 2016).

If genomic imprinting allows the separate role in resource allocation of maternal and paternal genes and thus potentially their separate evolutionary trajectories, then one may expect that genomic imprinting is under selective processes usually observed in the case of an evolutionary conflict, i.e. positive or balancing selection. Both in flowering plants and mammals, many studies have looked at selection footprints on the sequence of imprinted genes, especially searching for signs of positive selection, and have found some evidence (Smith and Hurst 1998; Spillane et al. 2007; Tuteja et al. 2019). However, these studies did not answer the question of whether genomic imprinting, as a molecular trait allowing parent-specific action, is under positive selection, but they rather showed that genomic imprinting preferentially arises in genes whose sequence is under positive selection.

One way to test whether genomic imprinting itself is under selection would be to consider the imprinted status as an inherited trait and to measure selection on this trait based on its segregation in natural populations. As a proxy for the imprinted status of a gene, one may use the insertion of transposable elements (TEs). TEs are mobile DNA sequences that constitute a substantial portion of the genome. They have been causally linked to the genomic imprinting of nearby genes (Li et al. 2023) due to the epigenetic reprogramming they undergo asymmetrically in male and female gametes (Calarco et al. 2012; Ibarra et al. 2012). Consistently, the presence/absence variation of TE insertions, or the epigenetic variation at TEs, is associated with the imprinted/not imprinted variation found in maize and *Arabidopsis thaliana*, respectively (Pignatta et al. 2014; Li et al. 2023).

One can thus assume that if the imprinting of a given gene is under positive selection, then the TE insertion responsible for it should also be under positive selection and increase in frequency in a population. This may be counterintuitive as TE insertions are rather depleted in and around genes, likely as a result of purifying selection due to their generally deleterious impact on genes and regulatory sequences (Bourque et al. 2018; Stritt et al. 2018). In fact, a recent work in *Brachypodium distachyon* has found that negative selection acts on TEs independently of their distance to genes (Horvath et al. 2024). This may explain why many TEs segregate in very low frequencies in natural populations (Stritt et al. 2018; Zhao et al. 2022). In addition, gene-neighboring TEs targeted by epigenetic silencing are under stronger negative selection than TEs that are not silenced in the species *Capsella grandiflora* (Horvath and Slotte 2017). This is likely due to the additional negative impact caused by the spread of the epigenetic modifications induced by the silencing of the TE insertion into neighboring genes and their regulatory regions (Hollister and Gaut 2009; Sammarco et al. 2022). In this context, as TEs require to be targeted by epigenetic silencing mechanisms to establish genomic imprinting, it is hard to imagine that positive selection acting on these silenced TEs could lead to their spread in a population, and yet, this remains a possibility.

In general, demographic events such as bottlenecks largely influence TE insertion frequencies (Stritt et al. 2018; Bourgeois et al. 2020). Demographic events are likely to interfere with selection and thus modify the accumulation of TEs, potentially leading to a “TE load” (Abascal et al. 2016; Stritt et al. 2018). For example, TE insertions segregating at lower frequencies in populations are more likely to be lost during a bottleneck due to genetic drift. Then, if beneficial alleles are lost by chance, selection cannot act on them. On the other hand, more common TE insertions can reach high frequency or even fixation by genetic drift, which could be misinterpreted as an effect of positive selection. Variation in the mating system also affects the efficacy of selection against TEs. Theory predicts that selfers show less effective selection against TEs than outcrossers due to a reduced population size and reduced probability of ectopic recombination (Montgomery et al. 1991; Bonchev and Willi 2018). Other authors have proposed opposite expectations, i.e. that selfing might facilitate the purging of deleterious recessive TE insertions (Wright and Schoen 1999; Morgan 2001). These demographic processes and life-history traits are likely to blur the impact of positive selection acting on TE insertions associated with genomic imprinting (if any). There are only a few whole-genome studies inferring the impact of demographic and selective processes on TE insertions, likely because it requires overcoming the technical difficulty of analyzing TE insertion patterns in multiple populations, but also requires a biological system where the demographic history is well characterized.

One biological system that fulfills such requirements is *Arabidopsis lyrata* L. This species originated in Central Europe, from which it colonized North America during the mid-Pleistocene (Schmickl et al. 2010). This was likely accompanied by a strong bottleneck, as North American populations are overall less diverse genetically compared with European ones (Schmickl et al. 2010). In North America, glacial refugia have been characterized in Pennsylvania (eastern clade) and Wisconsin (western clade). From this core area, postglacial recolonization occurred ∼20,000 years ago, leading to low genetic diversity and high genetic load in populations at the edges of the current distribution (Willi et al. 2018). At the edges of the distribution range, independent transitions to selfing have been reported, also accompanied by an increased genetic load (Foxe et al. 2010; Willi et al. 2018). A few studies have looked at the impact of the demographic history on TE accumulation in *A. lyrata*. One study found an increased number of TE insertions in the selfing populations compared with the outcrossing ones (Bonchev and Willi 2018). Another study compared Central European refugial populations (high genetic diversity) with colonized ones (Scandinavia and North America), and found an impact of bottlenecks on TE insertion patterns (Lockton et al. 2008). However, these studies used transposon display to support their conclusions, which is relatively low throughput and may not reflect whole-genome patterns.

In this study, we characterized the frequency and distribution pattern of TE insertions in the whole genome of *A. lyrata* populations from Europe and North America, with a particular focus on imprinted genes, and on populations having experienced different demographic events. Doing so, we aimed at (i) inferring selective processes acting on TE insertions, and especially on TEs targeted by epigenetic silencing; (ii) assessing the impact of successive demographic events on selection acting on TE insertions, based on their frequency and distribution after such events; and (iii) test whether TE-driven genomic imprinting is under positive selection.

## Results

### Population Bottlenecks and Postglacial Range Expansions have Shaped the Mutation Load Estimations in *A. lyrata*

**Fig. 1.**
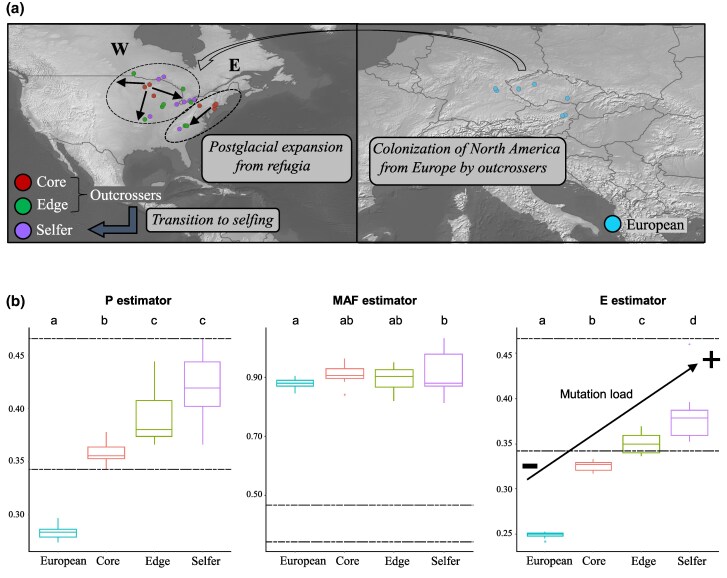
The demographic history of *Arabidopsis lyrata*. a) Species distribution maps of the North American (left) and the European (right) lineages of *A. lyrata*. The colonization event from Europe to North America and the subsequent transition to selfing are indicated. The populations belonging to the western (W) and eastern (E) clades described for the North American lineage are marked by circles. Within each clade, the direction of the postglacial expansion from the core areas is indicated by arrows. Population groups (European, core, edge, and selfer) are indicated by colors. b) Boxplots of estimated values on the four groups of populations analyzed (European, core, edge, and selfer) for the *P*, MAF, and *E* values (from left to right).

Previous works on the demographic and colonization history of *A. lyrata* in Europe and North America suggest successive bottlenecks and increases in mutation load, namely after (i) the colonization of North America from Europe (Schmickl et al. 2010), (ii) the postglacial expansion of North American populations from refugia to the edge ranges of distribution (Willi et al. 2018), and (iii) the transition to selfing in North America (Willi et al. 2022). We re-tested this hypothesis using metrics of the mutational load in these populations, as a foundation for our subsequent focus on the role of demographic events on TE accumulation. Our results show that the global genetic load estimator *E* (*E* = MAF × *P*, where minor allele frequency [MAF] = MAFns/MAFs and *P* = Pns/Ps) had significantly lower values for European populations of *A. lyrata* than for the North American ones, indicating an increase in mutation load in North American populations with respect to the European ones (Fig. 1, supplementary table S1, Supplementary Material online). Within the North Americans, the *E* estimator was lower for the core outcrossers than for the edge outcrossers and selfers (supplementary table S1, Supplementary Material online). This increase of the global genetic load estimator (*E*) was explained by an increase on the proportion (*P*) and on the frequencies (MAFs) of nonsynonymous polymorphic sites (Ps) compared with synonymous sites (Fig. 1).

Overall, these results confirm that several demographic processes have increased mutational load in *A. lyrata*: the colonization from Europe to North America, then the postglacial range expansion of the species in North America, and finally the transition to selfing in *A. lyrata*.

### The Role of the Demographic History of *A. lyrata* and Genetic Drift in the Number and Frequency of TEs

**Fig. 2.**
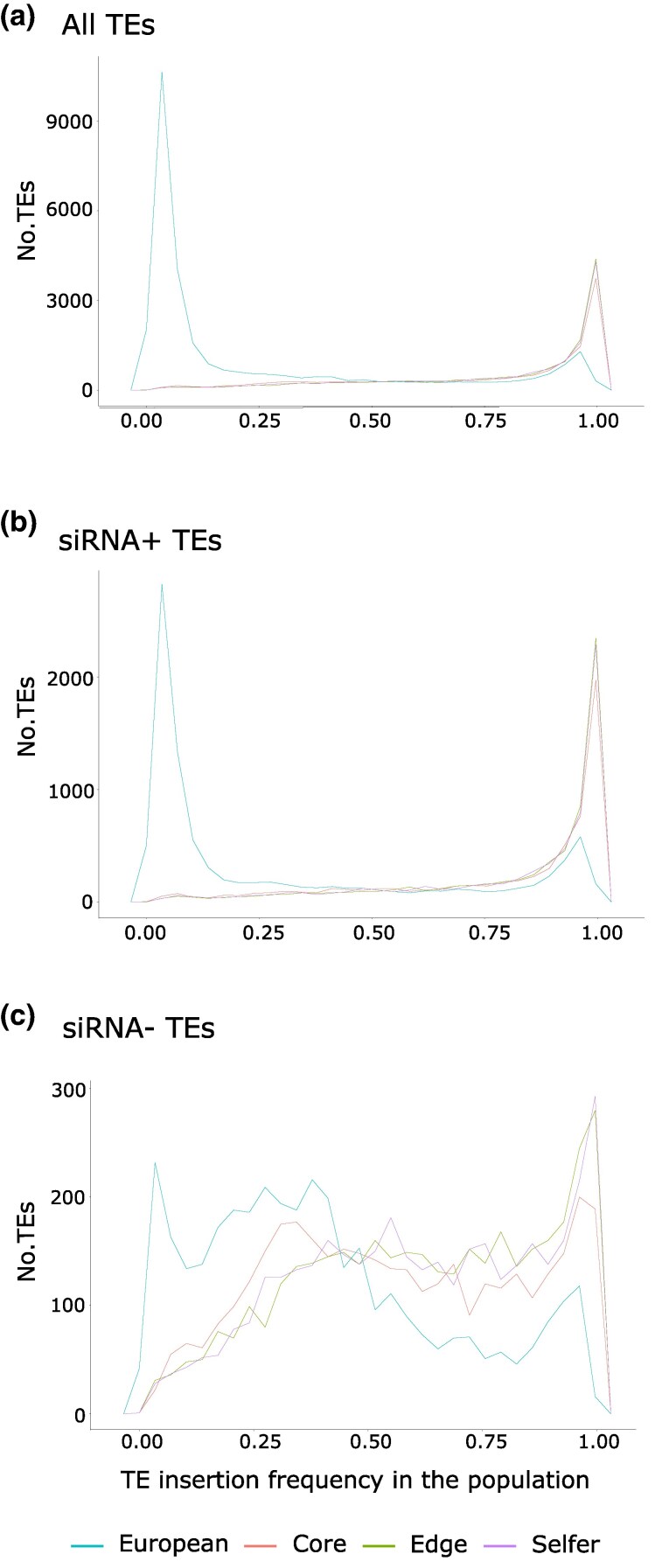
Number of TE insertions according to their frequency in the populations is shown for the different groups of *A. lyrata* analyzed in this study (European, core, edge, and selfer). a) Considering all TEs. b) Only TEs targeted by sRNAs are shown. c) Only TEs without sRNAs are shown.

The number of TE insertions identified in the European outcrossing populations was 30,056, while for the North American groups, the number of TE insertions significantly decreased: 13,406 for the core outcrossers, 14,013 for the edge outcrossers, and 13,995 for the selfers (Table 1). No major changes were observed in the percentage of TE orders or TE superfamilies between the population groups (Table 1; supplementary fig. S1, Supplementary Material online). The decrease in the percentage of non-LTRs observed in the European group was mainly caused by TEs classified as LINEs. The huge rise in the number of TE insertions identified in the European outcrossers with respect to North American selfers and outcrossers was mainly due to rare TE insertions (Fig. 2a). Effective population sizes are estimated to be smaller in the North American lineage of *A. lyrata* than in populations from Europe (Lockton et al. 2008; Ross-Ibarra et al. 2008). After a bottleneck and when effective population sizes are small, TEs segregating in low frequencies are likely to be lost, while TEs segregating at medium frequencies may reach fixation (Bourgeois and Boissinot 2019). Accordingly, we observed that the average frequency of TE insertions is higher in the North American populations, in line with the successive bottlenecks occurring in this *A. lyrata* lineage, and the comparatively lower population sizes. When looking at TE frequencies (Table 1 and Fig. 2a), we observed that the number of fixed TE insertions was high for all the North American groups (∼62% to 68%), while only ∼14% of TE insertions were fixed in the European outcrossing group. Conversely, European outcrossers have a very high number of rare TE insertions (∼69%) compared with the North American groups for which the number of rare TE insertions varied between ∼5% and 7% for selfers and outcrossers. The number of TE insertions classified as frequent (those showing 0.25 to 0.75 frequency values) was more similar among European and North American populations and represented ∼17% and ∼27% to 31% of the total in European and North American groups, respectively (Table 1). The same trend was confirmed for the five TE orders (i.e. DNA transposons, LTR, non-LTR, helitrons, and other retrotransposons; supplementary table S2, Supplementary Material online). Accordingly, the median frequency of TEs identified in Europeans was 0.067. Within North American groups, the median frequency of TEs was 0.884 in core outcrossers, 0.912 in edge outcrossers, and 0.904 in selfers. Percentages of TEs targeted by sRNAs and thus, considered as silenced, were more abundant than nonsilenced TEs for all groups of populations of *A. lyrata* (supplementary table S2, Supplementary Material online). Accordingly, the trend we observed showing a majority of rare TEs in Europeans and a majority of fixed TEs in North Americans was likely driven by silenced TEs, which showed the same frequency pattern (Fig. 2b). To the contrary, more nonsilenced TEs rather showed intermediate frequencies for all population groups (Fig. 2c).

**Table 1:** Total number of TE insertions identified in each population group of *A. lyrata* analyzed in this study (European, core, edge, and selfer)

Number of TEs	European outcrossers		North American core outcrossers		North American edge outcrossers		North American selfers	
	*n*	%	*n*	%	*n*	%	*n*	%
Total	30,056		13,406		14,013		13,995	
*According to frequency*								
Rare	20,826	69.29	944	7.04	741	5.29	744	5.32
Frequent	5,084	16.92	4,075	30.40	3,800	27.12	3,962	28.31
Fixed	4,146	13.79	8,387	62.56	9,472	67.59	9,289	66.37
*According to order*								
DNA transposons	14,000	46.58	5,723	42.69	6,208	44.30	6,331	45.24
LTR	7,866	26.17	3,260	24.32	3,206	22.88	3,121	22.30
Non-LTR	4,608	15.33	2,681	19.99	2,705	19.30	2,691	19.23
Helitrons	3,534	11.76	1,696	12.65	1,849	13.19	1,809	12.93
Retrotransposons	48	0.16	46	0.34	45	0.32	43	0.31
*According to family*								
Helitron	3,534	13.32	1,696	14.48	1,849	15.20	1,809	14.84
MuDR	3,978	15.00	2,023	17.28	2,081	17.11	2,108	17.30
hAT	2,065	7.79	823	7.03	876	7.20	897	7.36
TIR_MITE	1,982	7.47	758	6.47	848	6.97	855	7.02
Harbinger	1,399	5.27	489	4.18	520	4.27	539	4.42
CACTA	1,220	4.60	607	5.18	624	5.13	610	5.01
Mariner	1,030	3.88	276	2.36	414	3.40	475	3.90
Tase	277	1.04	137	1.17	131	1.08	131	1.08
Copia	3,761	14.18	1,397	11.93	1,411	11.60	1,348	11.06
Gypsy	3,207	12.09	1,589	13.57	1,520	12.50	1,495	12.27
TRIM_LARD	898	3.39	274	2.34	275	2.26	278	2.28
LINE	3,197	12.05	2,029	17.33	2,040	16.77	2,026	16.63
SINE	1,411	5.32	652	5.57	665	5.47	665	5.46
NA	2,097	7.91	656	5.60	759	6.24	759	6.23

Number of TE insertions identified in each population group according to their frequency (rare, frequent, or fixed). Number of TE insertions identified in each population group according to the TE order (DNA transposons, LTR, non-LTR, helitrons, and other retrotransposons). Number of TE insertions identified in each population group according to TE superfamilies.

To rule out technical biases explaining these results, we used a European genome reference (*A. lyrata* ssp. *petraea*), and found very similar TE insertion patterns independently of the reference genome we used (supplementary fig. S2 and table S3, Supplementary Material online). Also, long-read sequencing data of three North American individuals showed a high rate of homozygosity for structural variants (SVs; supplementary fig. S3, Supplementary Material online), consistent with a large proportion of TEs fixed in these populations. In addition, SVs detected as deletions compared with the reference genome expectedly matched TE insertions of lower frequencies in the populations (supplementary fig. S4, Supplementary Material online). A similar comparison with SV detected as insertions yielded only a few TEs (supplementary fig. S4, Supplementary Material online), likely because TE insertions present both in the reference genome and the three individuals are not reported as SVs and are thus not crossable with the population data.

### Impact of the Type of TEs and Their Silencing on Their Accumulation Around Genes

**Fig. 3.**
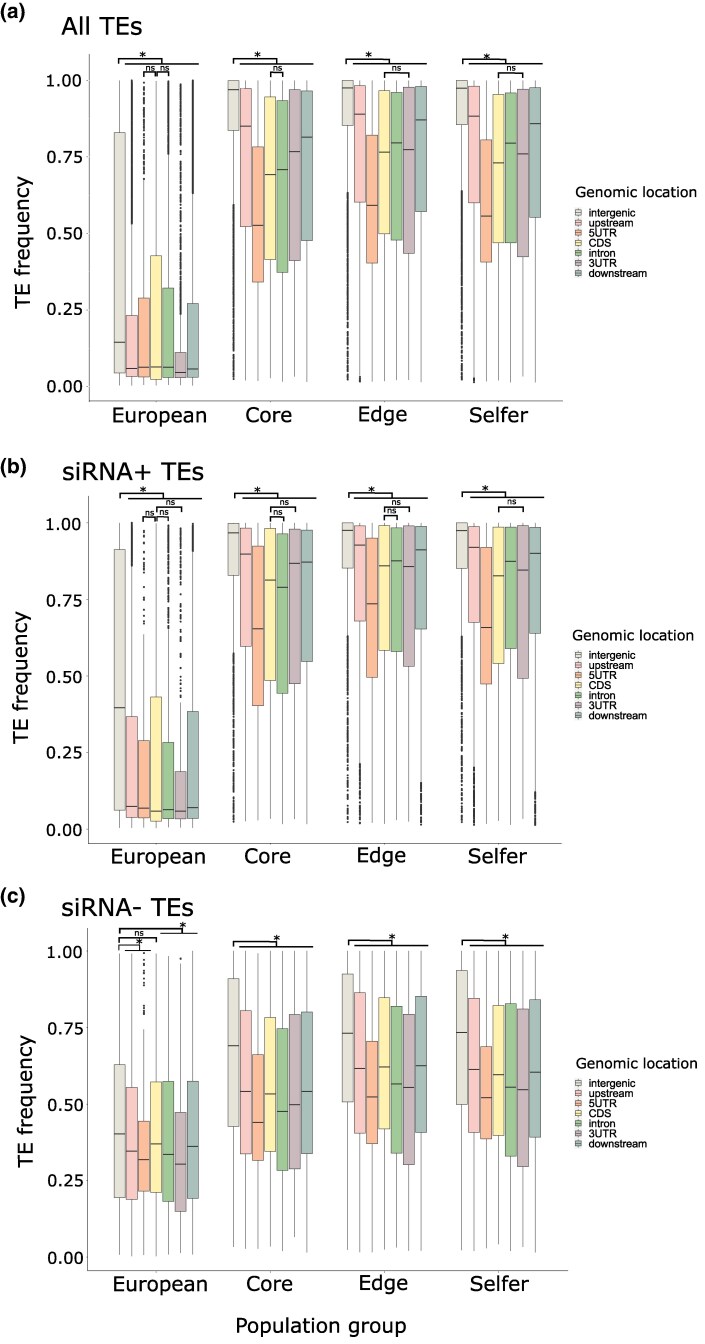
Frequency of TE insertions in the different population groups of *A. lyrata* analyzed (European, core, edge, and selfer) is shown by the genomic location in which TEs are associated (intergenic, upstream, 5′ UTR, CDS, intron, 3′ UTR, and downstream). a) Considering all TEs. b) Only TEs targeted by sRNAs are shown. c) Only TEs without sRNAs are shown. An asterisk indicates a significant median difference by permutation test (*P* < 0.05).

A common assumption is that TE insertions are deleterious, particularly when located in and around genes, and especially if they are targeted by epigenetic mechanisms (Hollister and Gaut 2009). Here, we tested this hypothesis by looking at the frequency and epigenetic silencing status of TE insertions around genes (Table 2). For all groups of populations analyzed (North Americans and Europeans), TE insertions located within the gene body and 2 kb flanking regions showed a significantly lower frequency than those found in intergenic regions (Fig. 3a; supplementary table S4, Supplementary Material online). This trend was found for all TE orders (supplementary fig. S5, Supplementary Material online).

**Table 2:** Number and percentage of TE insertions identified in each population group of *A. lyrata* analyzed (European, core, edge, and selfer) according to the genomic location in which TEs are associated (intergenic, upstream, 5′ UTR, CDS, intron, 3′ UTR, and downstream)

Number of TEs	European outcrossers		North American core outcrossers		North American edge outcrossers		North American selfers	
	*n*	%	*N*	%	*N*	%	*n*	%
*According to genomic location*								
Intergenic	4,645	9.87	3,531	18.80	3,647	18.71	3,610	18.45
Upstream	14,851	31.57	5,163	27.49	5,535	28.40	5,645	28.86
5′ UTR	1,382	2.94	501	2.67	503	2.58	488	2.49
CDS	6,087	12.94	2,471	13.16	2,417	12.40	2,443	12.49
3′ UTR	1,887	4.01	384	2.04	374	1.92	350	1.79
Intron	3,790	8.06	1,524	8.11	1,526	7.83	1,507	7.70
Downstream	14,406	30.62	5,208	27.73	5,490	28.17	5,519	28.21
Total	47,048	…	18,782	…	19,492	…	19,562	…

In particular for European outcrossing populations, the lowest frequency of TEs was of those located in the 3′ UTR, upstream, and downstream regions of genes, although the frequency of TEs annotated in the coding regions (CDSs) was not much higher than those (Fig. 3a; supplementary table S4, Supplementary Material online). When focusing on silenced TEs, we also found a significantly higher frequency of silenced TEs in intergenic regions than within the gene body and the 2 kb flanking region (Fig. 3b; supplementary table S4, Supplementary Material online). This was also true for nonsilenced TEs, except for the CDS region (supplementary table S4, Supplementary Material online). Moreover, the European outcrossers did not show significantly different frequencies among TEs located within different genic regions, regardless of whether they were targeted by silencing or not (Fig. 3b and c; supplementary table S4, Supplementary Material online).

In all North American groups of populations (core, edge, and selfer), we also found a significantly higher frequency of TEs in intergenic regions than within the gene body and the 2 kb flanking region, independently of whether TEs were affected by silencing or not (Fig. 3b and c; supplementary table S4, Supplementary Material online). TE insertions located inside the 5′ UTR showed the lowest frequency, followed by those annotated within the coding regions (CDS). It should be pointed out that TE frequencies in CDS regions were not significantly different from those found inside the 3′ UTR or intron regions in some groups of populations (supplementary table S4, Supplementary Material online). For simplicity, these nonsignificant differences are only shown in Fig. 3a and b. Most of the frequencies of nonsilenced TEs observed in distinct genic regions were not significantly different from each other (supplementary table S4, Supplementary Material online).

We then tested whether the expression pattern (ubiquitous vs. endosperm-specific) of genes could influence TE insertion frequencies, under the assumption that TE insertions near a ubiquitous gene may have a more pleiotropic, and therefore deleterious, effect. Indeed, we found that for edge outcrossing and selfing North American population groups, TE frequencies around ubiquitous genes were significantly lower than for endosperm-specific genes (supplementary fig. S6 and table S5, Supplementary Material online). On the contrary, European and North American core outcrossing populations showed no significant frequency differences between TEs located around ubiquitous and those near endosperm-specific genes (supplementary fig. S6 and table S5, Supplementary Material online). No significant differences were found between any group when looking separately at silenced and nonsilenced TEs (supplementary fig. S7 and table S5, Supplementary Material online).

Overall, our results show that TE frequencies are lower around and inside genes compared with nongenic regions, independently from the fact that TEs are targeted by epigenetic silencing or not. The frequency of TEs within the 5′ UTR region was significantly lower in all North American groups particularly when TEs were silenced. Coding parts of genes did not always show lower frequencies of TEs compared with introns or flanking regions. Endosperm-specific genes showed higher TE frequencies than ubiquitous genes only in the North American population groups of *A. lyrata* that were located at the edge of the distribution or that have shifted to selfing.

### The Impact of Genomic Imprinting on the Frequency of TE Insertions

Theoretical predictions suggest that genomic imprinting itself, as a molecular trait, may show fitness benefits and thus be under positive selection. We tested this hypothesis by measuring TE frequencies around imprinted genes (identified by Klosinska et al. 2016) and comparing them to the frequency of TEs that are not expected to be under positive selection (i.e. TEs around endosperm-specific and ubiquitous genes). For all European and North American populations, the frequency of TEs associated with MEGs and those associated with PEGs were not significantly different when compared with those TEs associated with endosperm or ubiquitous genes (supplementary fig. S6 and table S5, Supplementary Material online). Compared with TEs found in intergenic regions, the TEs associated with MEGs did not show a significant difference in their frequency, while the frequencies of TEs associated with endosperm-specific and ubiquitous genes were significantly lower (supplementary fig. S6 and table S5, Supplementary Material online). When looking at the frequencies of TEs associated with PEGs, they were not significantly different from those found in intergenic regions in European populations, while in North American populations, the frequencies of TEs associated with PEGs were significantly lower (supplementary fig. S6 and table S5, Supplementary Material online).

When analyzing silenced versus nonsilenced TEs, we looked at the frequencies of MEGs and PEGs together in order to avoid a very low sample size. Similarly, for all groups of populations analyzed, we did not find significant differences when directly comparing the frequency of TEs associated with imprinted, endosperm, and ubiquitous genes (supplementary fig. S7 and table S5, Supplementary Material online). When comparing to intergenic regions, we found that the frequencies of silenced TEs were significantly different when they were associated with endosperm-specific or ubiquitous genes, but not when they were associated with imprinted genes (supplementary fig. S7B and table S5, Supplementary Material online).

**Fig. 4.**
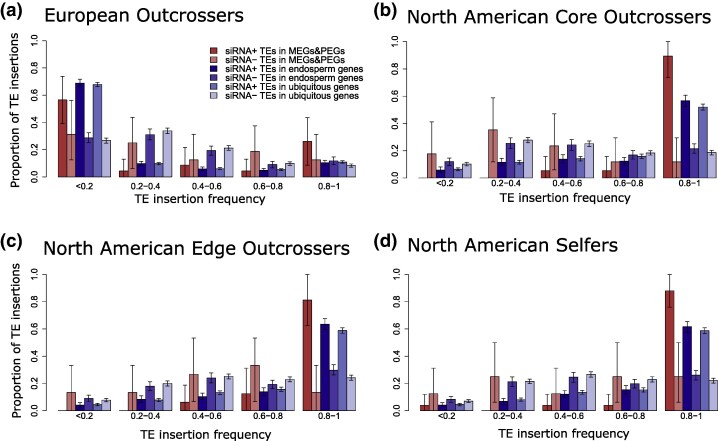
Insertion frequency spectra of silenced and nonsilenced TE insertions associated with imprinted, endosperm-specific, and ubiquitous genes for each group of populations of *A. lyrata*: a) European outcrossers; b) North American core outcrossers; c) North American edge outcrossers; and d) North American selfers. Ninety-five percent confidence intervals from 1,000 bootstraps of each TE category are shown. MEGs and PEGs: maternally and paternally expressed genes, respectively.

We then computed the insertion frequency spectra (IFS) of silenced and nonsilenced TEs associated with the different categories of genes (i.e. imprinted, endosperm-specific, and ubiquitous genes) in order to distinguish traces of selective forces acting on TE insertions depending on their epigenetic silencing. In Europeans, we found a significantly higher proportion of rare silenced than nonsilenced TEs when they were associated with endosperm or ubiquitous genes (Fig. 4), suggesting that silenced TEs associated with endosperm and ubiquitous genes are under stronger purifying selection than silenced TEs associated with imprinted genes. However, this was not true for any of the North American populations of *A. lyrata* analyzed in our study. For core, edge, and selfer North American groups, we found a higher proportion of fixed silenced TEs when those are associated with imprinted genes than when TEs are associated with ubiquitous genes (Fig. 4). The proportion of fixed silenced TEs associated with imprinted genes was also higher than those associated with endosperm genes in core and selfer North American populations. This pattern was not found for nonsilenced TEs.

## Discussion

### Mutation Load is Driven by Population Bottlenecks and Postglacial Range Expansions in *A. lyrata*

Previous studies have compared the mutation load of core/edge outcrossing and selfing North American populations of *A. lyrata* (Willi et al. 2018). However, mutation load estimations of European populations of the species have not been previously reported. For the first time, we compared mutation load estimations between the North American and the European lineages of *A. lyrata*. Our analyses showed that the mutation load is higher in the bottlenecked populations from North America than in the European populations that served as source populations (Fig. 1) according to the colonization history of *A. lyrata* (Ross-Ibarra et al. 2008; Schmickl et al. 2010). Additionally, the demographic history of *A. lyrata* in North America has been strongly affected by ancient postglacial expansion from the eastern and western core areas toward the edges of the distribution after the last glacial maximum (LGM). Our mutation load estimations (Fig. 1; supplementary table S1, Supplementary Material online) are higher for edge than for core outcrossing populations of *A. lyrata*, and are in agreement with the scenario of postglacial colonization as previously reported (Willi et al. 2018). Finally, the transition to selfing in the North American lineage of *A. lyrata* has occurred at the edge distribution of the species (Griffin and Willi 2014). Our estimates support a higher mutation load in selfing populations than core and edge outcrossing ones in *A. lyrata* (Fig. 1; supplementary table S1, Supplementary Material online), consistent with previous studies (Willi et al. 2018).

### Demographic Events and Purifying Selection have Shaped the Transposon Load in *A. lyrata*

The number and frequency of TE insertions in natural populations can be influenced by demographic events and natural selection (Bourgeois and Boissinot 2019). For the first time, taking into account its complex demographic history, we examined the TE landscape of *A. lyrata* using sequence data from the entire genome. In the case of TEs, insertions targeted by epigenetic silencing and located in genic regions may be the most deleterious for the fitness of their host because they can interfere with the expression of genes and regulatory sequences, or increase the risk of ectopic recombination (Hollister and Gaut 2009; Bonchev and Willi 2018). In our study, we generally found for all groups of *A. lyrata* that TE insertions showed a significantly lower frequency when they were located nearby or within genes than when they were found in intergenic regions (Fig. 3a; supplementary table S4, Supplementary Material online). This is consistent with previous studies showing the action of purifying selection in TE dynamics in *Arabidopsis* (Hazzouri et al. 2008; Lockton et al. 2008), or in other plant species such as *B. distachyon* (Stritt et al. 2018) or in vertebrates (Ruggiero et al. 2017; Xue et al. 2018). This exclusion from genic regions was especially strong for TEs targeted by small RNAs, and less so for untargeted TEs, suggesting that the epigenetic silencing of TEs is particularly deleterious as it may interfere with gene expression (Hollister and Gaut 2009; Horvath and Slotte 2017). These results agree with the expectation that purifying selection acts against TEs that are inserted into or near genes, preventing the fixation of those TEs that have more deleterious effects on the host.

Interestingly, the patterns greatly varied between North American and European groups of *A. lyrata* populations, suggesting a strong interference of selective processes by demographic events. On one hand, genetic drift may lead to the loss of rare TE insertions and the increased frequency/fixation of more frequent TE insertions by mere stochasticity (Lynch and Conery 2003). Consistently with an impact of genetic drift in TE insertions driven by the colonization history of *A. lyrata*, we observed a high proportion of fixed TEs in all North American groups of *A. lyrata* populations, and very few rare TE insertions. In addition, population size is an important factor in TE evolution because the elimination of deleterious mutations by the effect of purifying selection decreases when an effective size population is small (Lynch and Walsh 2007). In detail, with increased inbreeding in bottlenecked populations, one may expect the purge of highly deleterious TEs, which are not masked anymore by heterozygosity, and the accumulation of mildly deleterious TEs (“transposon load”; Lynch and Conery 2003). As expected for highly deleterious mutations, most silenced TEs segregate at low frequencies in nonbottlenecked European populations in our study. These rare insertions of silenced TEs appear to be lost in North American populations, and this loss is found to be stronger in edge outcrossers and selfer populations of North America than in core outcrossers, which is consistent with the purging effect of inbreeding on highly deleterious mutations (Wright and Schoen 1999; Boutin et al. 2012).

The other side of the inbreeding coin is the accumulation of mildly deleterious TE insertions or “TE load.” This is likely to explain the increased frequencies of TEs from European populations to North American ones, and from the core North Americans to the edge ones in our study, paralleling the consecutive decreases in effective population size (Ross-Ibarra et al. 2008; Willi et al. 2018). In our study, North American edge outcrossers showed the highest proportion of fixed TEs. Under this scenario, one may expect TEs located nearby genes and that are not targeted by epigenetic silencing to be mildly deleterious, and thus to accumulate and increase in frequency in bottlenecked populations. Indeed, we found these TE insertions to have higher frequencies in North American populations compared with European ones. However, in North American populations, silenced TEs, supposedly highly deleterious, showed higher frequency than nonsilenced TEs, even those located in genic regions, which contradicts our expectations. This pattern may nevertheless be explained by the loss of most rare silenced TEs in North American populations of *A. lyrata* during the colonization process, leaving only the silenced TE insertions that were in high frequency before the colonization and therefore, likely to be the less deleterious ones. Overall, the North American *A. lyrata* populations seem to carry a high TE load, potentially harming their general fitness and limiting their expansion and adaptability in the long term. Alternatively, these TEs could serve as an important source of genetic variation, providing a great opportunity for TE-mediated evolutionary changes and local adaptation (Casacuberta and González 2013) in these populations with high levels of isolation and reduced population size.

Although theoretical expectations predict a loss of TE copies after a shift to selfing (Boutin et al. 2012), selfers of *A. lyrata* showed a similar total number of TEs compared with outcrossers located at the edge of the distribution in our study. Nevertheless, our results are consistent with a previous study in *A. lyrata* (Bonchev and Willi 2018), in which a higher TE copy number and a higher mean TE allele frequency were found in selfing populations compared with outcrossers, again inconsistent with predictions of TE loss in selfers. The frequency estimations of SI (self-incompatible) plants within populations of *A. lyrata* (Willi and Määttänen 2010) suggest that outcrossing events might occur in selfing populations of *A. lyrata* (Foxe et al. 2010), possibly delaying the loss of TEs. Altogether, our results support the demographic history as one of the major factors shaping the TE population numbers and frequencies of *A. lyrata*, which is congruent with previous studies based on TE-display band data and a smaller sampling (Lockton et al. 2008; Bonchev and Willi 2018). Our results expand our understanding of the molecular processes influencing selection against TEs in *A. lyrata*, including their epigenetic silencing and their proximity to genes.

### Genomic Imprinting Established by TEs is Likely Under Positive Selection

To test whether positive selection could act on TE causing genomic imprinting, we monitored the frequency of silenced and nonsilenced TEs around previously identified imprinted genes (Klosinska et al. 2016), under the assumption that the TE insertions responsible for the imprinting of these genes would spread in populations if beneficial. As TEs require being targeted by epigenetic silencing mechanisms to establish genomic imprinting, we expected that positive selection could be acting preferentially on silenced TEs. In our study, we did not find significant differences when the frequency of silenced TEs associated with imprinted genes was directly compared with endosperm or ubiquitous genes (supplementary figs. S6, S7, and table S5, Supplementary Material online). One might think at first that these results contradict our expectations. Nevertheless, when the TE frequencies were compared with nongenic regions, we only found significant lower frequencies on the TEs associated with endosperm or ubiquitous genes, suggesting that purifying selection was acting more strongly on those than on TEs associated with imprinted genes, especially MEGs. In addition, for all North American groups of populations, we found a significantly higher proportion of fixed silenced TEs when those are associated with imprinted genes than when TEs are associated with ubiquitous genes (Fig. 4). However, we cannot rule out that the increase in frequency of TEs associated with imprinted genes had also been affected by the strong genetic drift observed in North American populations, but not European ones. In fact, the increased frequency of silenced TEs associated with imprinted genes was also found in European populations, for which we did not see a sign of TE load. This increase in frequency of imprinting-associated TEs, however, was not significant, and it did not go up to fixation as observed for North American populations according to the Insertion Frequency Spectra analysis (Fig. 4). Nevertheless, if the patterns we observe are driven by genetic drift, the effect will be expected to be the same for all genes, independently of their genomic location or if they are imprinted or not. Overall, our results are consistent with the expectation that genomic imprinting, as a molecular trait, would provide an advantage, possibly under a parental conflict scenario (Batista and Köhler 2020), and thus imprinted alleles would spread or even get fixed in natural populations.

## Conclusions

In this study, we propose that purifying selection acts against epigenetically silenced TEs, especially if they are inserted in genic regions. Successive bottlenecks in *A. lyrata* seem to have increased its TE load, including the load of epigenetically silenced TEs.

Despite the widespread action of purifying selection and demographic events, silenced TEs increased in frequency when they were linked to imprinted genes. This suggests that in *A. lyrata*, genomic imprinting could have been established through positive selection acting on silenced TEs, potentially as a response to parental conflict.

## Materials and Methods

### Pool-Sequencing Data

We leveraged Pool-Seq data from populations of European and North American lineages of *A. lyrata* generated by Guggisberg et al. (2018) and Willi et al. (2018), respectively. We downloaded paired-end reads from all 7 populations sampled in Europe (30 individuals per population, all outcrossing). Within North American populations, we selected 8 selfing, 8 core outcrossing, and 8 edge outcrossing populations of *A. lyrata* (25 individuals per population). The eight core populations were those showing the lowest geographic distance to the area of the LGM refugia for the species, according to the results obtained by Willi et al. (2018). The eight edge populations corresponded to those located at the largest distance from the LGM refugia. The total number of analyzed populations was 31 (supplementary table S6, Supplementary Material online). Hereafter, we will refer to the different groups of populations as: European, core, edge, and selfer.

### Long-read Sequencing Data

Additionally, nanopore sequencing was used to generate long-read sequences for three different individuals from North America (ON4, selfer, IN1, ON5, and edge outcrossers) in order to validate the identification of TE insertions from Pool-Seq data. To this aim, 2 g of fresh leaves were collected from three different individuals from North America (one selfer, two edge outcrossers) and flash-frozen. High-molecular-weight genomic DNA was extracted, as described in Belser et al. (2018). Long-read sequences were obtained by Oxford Nanopore Technologies (ONT) flowcells v9. To polish the contigs obtained by long-read sequencing, Illumina data were also generated (with no step of PCR amplification to minimize sequencing bias). For Nanopore library preparation, the smallest genomic DNA fragments were first eliminated using the Short Read Eliminator Kit (Pacific Biosciences). Libraries were then prepared according to the protocol 1D Native barcoding genomic DNA (with EXP-NBD104 and SQK-LSK109) provided by ONT. Genomic DNA fragments were repaired and end-prepped with the NEBNext FFPE DNA Repair Mix and the NEBNext Ultra II End Repair/dA-Tailing Module (New England Biolabs [NEB]). Barcodes provided by ONT were then ligated using the Blunt/TA Ligase Master Mix (NEB). Barcoded fragments were purified with AMPure XP beads (Beckmann Coulter), and then pooled, and ONT adapters were added using the NEBNext Quick Ligation Module (NEB). After purification with AMPure XP beads (Beckmann Coulter), each library was mixed with the sequencing buffer (ONT) and the loading beads (ONT) and loaded on a PromethION R9.4.1 flow cell. In order to maintain the translocation speed, flow cells were refueled with 250 µL Flush Buffer when necessary. Reads were basecalled using Guppy version 3.2.10, 4.0.11, 5.0.12, 5.0.13, 5.0.16, or 5.1.12. The Nanopore long reads were not cleaned, and raw reads were mapped to the *A. lyrata* v2.1 reference genome (Hu et al. 2011) using Minimap2 (v2.24-r1122; Li 2018 ) with the “map-ont” mode. Sniffles (v2.0.7; Smolka et al. 2024) was then used with the A mode (general) using default parameters to predict SVs in each individual as described here (https://github.com/fritzsedlazeck/Sniffles). Insertion and deletion variants of <100 kb and with all default Sniffels filters of quality passed were kept for downstream analysis. Using bedtools (v2.29; Quinlan and Hall 2010), we retain SVs that intersected with observed TE polymorphisms data (allowing up to 150 bp distance). We then tested for differences in TE polymorphic frequency between TEs intersected with SVs and TEs nonintersected with SVsSV-associated loci and non-SV-associated loci, employing the nonparametric Wilcoxon test with R's ggpubr package (R version 4.3.3, ggpubr v0.6).

### Mutation Load Estimations

#### Read Mapping and Variant Calling

Raw reads were mapped to the *A. lyrata* v2.1 reference genome (cultivar MN47, NCBI taxonomy ID: 59689; Hu et al. 2011) using Bowtie2 v2.2.4 (Langmead and Salzberg 2012). The quality of the mapping of the paired-end reads was checked using samtools v0.1.19 (Li et al. 2009; supplementary table S1, Supplementary Material online). Files were then converted to BAM format using samtools v0.1.19 (Li et al. 2009). The SNPs in these regions were called using the Genome Analysis Toolkit v. 4.0.12 (GATK; DePristo et al. 2011) with the option GVCF after variant filtration using a quality score ≥30. We modified the ploidy parameter in GATK to consider all haplotypes in each population (ploidy = 50 and 60 which corresponded to 25 and 30 diploid individuals for the populations of North America and Europe, respectively). For each sample independently, we computed the distribution of coverage depth across position using samtools depth (Li et al. 2009). We considered only biallelic or monomorphic sites with a depth coverage ≥25 or 30 for the North American and European populations, respectively, and <500, as it is described in Willi et al. (2018). After filtration, we calculated the mean coverage of each sample (supplementary table S1, Supplementary material online).

#### Quantifying the Genetic Load

To estimate the genetic load in each population, we first annotated the 0- and 4-fold degenerate sites in the *A. lyrata* v2.1 reference genome (Hu et al. 2011) using the script NewAnnotateRef.py (Williamson et al. 2014). Because all mutations at 0-fold degenerate sites alter the sequence of the encoded protein (nonsynonymous variants), we assumed that these mutations are deleterious (neglecting the rare cases where balancing selection could favor amino acid changes). In contrast, mutations at the 4-fold degenerate sites never alter the encoded amino acid (synonymous variants), so we used them as neutral references. For the sake of simplicity, we discarded mutations on the 2- or 3-fold degenerate sites.

We estimated the proportion of nonsynonymous variants (Pns) as the number of polymorphic 0-fold degenerate sites divided by the number of covered 0-fold degenerate sites. However, a decrease in the proportion of Ps is also expected for neutral sites (Tajima 1989). Thus, we also estimated the proportion of synonymous Ps based on 4-fold degenerate sites as a proxy of the general effect of genetic drift. Then, we estimated the ratio *P* = Pns/Ps to compare the effect of bottleneck in the proportion of Ps. The Pns/Ps ratio is an indicator of the efficacy of purifying selection, with higher values suggesting lower efficacy, and thus higher mutational load (Lohmueller et al. 2008; Willi et al. 2018).

Another expected effect of a bottleneck is the increase of the chance of fixation of some deleterious alleles. To study this effect, we estimated the MAF of each population under the assumption that the deleterious allele is the allele that is present in lower frequency at each position. We used the depth coverage of each SNP in each position as a proxy of the allelic frequency in the population. We estimated the mean minor allele frequency obtained for the nonsynonymous (MAFns) and synonymous variants (MAFs) as the minor allele frequency obtained for the 0- and 4-fold degenerate sites, respectively. Then, we estimated the ratio MAF = MAFns/MAFs. We expected an increase of this ratio in North American *A. lyrata* populations due to the effect of the bottleneck associated with the colonization history of the species. In fact, deleterious mutations are expected to be maintained by selection in low frequencies in the population. However, small population sizes hamper selection, and deleterious mutations can increase their frequencies if these are retained during the bottleneck (Kirkpatrick and Jarne 2000). Finally, we estimated the global genetic load by population as the interaction between the proportion and the frequencies of nonsynonymous mutations, following the ratio *E* = (Pns × MAFns)/(Ps × MAFs). In order to test whether the values obtained for these estimators were significantly different among groups of populations, we performed permutation tests using 10,000 replicates for each pair of groups of populations compared, using a custom R code (https://github.com/leveveaudrey/permutation-test-).

### Identification of TE Insertion Sites

In order to identify TE insertion sites, we followed the recommendations in Kofler et al. (2016) and Horvath and Slotte (2017) for running PoPoolationTE2. We mapped the reads to a TE-merged reference genome using *bwa bwasw* (Li and Durbin 2009). Since *bwasw* does not support mapping paired-end reads, we restored paired-end information using PoPoolationTE2 (Kofler et al. 2016). The TE-merged reference was generated by merging the *A. lyrata* reference genome (in which the TE sequences were previously masked using bedtools) and the corresponding TE sequences. To achieve this, we used the North American *A. lyrata* v2.1 reference genome (Hu et al. 2011) and the TE annotation for the same reference provided by Legrand et al. (2019). In order to ensure that the use of this reference genome did not introduce any bias, we also identified the TE insertions in all groups of populations using the European *A. lyrata* ssp. *petraea* genome assembly with the corresponding TE library (cultivar MJ09-11; Bramsiepe et al. 2023). We used PoPoolationTE2 for the identification and the estimation of TE insertion frequencies. For both genomes, we used a joint mode analysis independently for each group of *A. lyrata* populations (European, core, edge, and selfer) following the recommendations for these analyses in Kofler et al. (2016) and Horvath and Slotte (2017). We filtered out TEs showing an average coverage <10, and a maximum frequency of other TEs and/or SVs of 0.2. Using this method, both annotated and novel (i.e. not annotated insertions in the reference genome) TE insertions were identified (Kofler et al. 2016). In order to get a similar power to identify TE insertions within as well as between groups of populations, it is usually recommended to subsample the pileup to a uniform physical coverage before identifying signatures of TE insertions (Kofler et al. 2016). However, we noticed that subsampling the physical coverage in our data resulted in a dramatic loss of TE insertions in Europeans even when applying low coverage values (supplementary tables S3 and S7, Supplementary Material online). In order to check whether the subsampling was favoring/biasing the identification of low- or high-frequency TE insertions, we plotted the site frequency spectrum of TEs identified in each group of populations using both approaches, i.e. with and without applying the subsampling. We confirmed that applying, for example, a subsampling of a minimum coverage of ten removed the majority of TE insertions that were identified in a low frequency in the European populations (supplementary fig. S8, Supplementary Material online). Given this issue, we performed the identification and estimation of TE insertion frequencies without applying any subsampling. Instead, we downsampled all BAM files to a uniform genome coverage before running PoPoolationTE2 (supplementary table S6, Supplementary Material online) in order to avoid the bias in the power to identify TE insertions within as well as between groups of populations. Following this approach, we confirmed that no significant correlation was found between coverage of BAM files and number of identified TEs (supplementary fig. S9, Supplementary Material online) and we avoided the loss of rare TE insertions in the European group (supplementary fig. S8, Supplementary Material online). We kept only those TE insertions identified in chromosome-size scaffolds 1 to 8. According to their frequency, we classified TE insertions as rare (<0.25), frequent (0.25 to 0.75), and fixed (>0.75).

### Identification of TEs Targeted by 24-Nucleotide Small RNAs

In order to distinguish between silenced versus nonsilenced TE insertions, we identified which TE insertions were targeted by siRNAs and which ones were not. To this aim, we used small RNA sequencing data from leaves (3 samples) and flowers (4 samples) of *A. lyrata* from previous studies (Ma et al. 2010; supplementary table S8, Supplementary Material online). In cases when the siRNAs were available only as raw reads, gsat (https://github.com/MikeAxtell/gsat) was used to trim these reads using default settings; otherwise, already trimmed reads were downloaded and used.

The siRNA reads were mapped to the *A. lyrata* genome assembly (Hu et al. 2011) using ShortStack (https://github.com/MikeAxtell/ShortStack; Johnson et al. 2016) with default settings, allowing for one mismatch, restricting the analyses to 20 to 25 nt siRNAs (–dicermin 20 –dicermax 25) and using *A. lyrata* TE annotation (Legrand et al. 2019) to count the siRNAs mapping to annotated TEs. We then used the 24-nt siRNA and did their RPM normalization to all mapped siRNA (summary statistics in supplementary table S8, Supplementary Material online). We considered TEs with average RPM = 0 to not be targeted by sRNAs (nonsilenced). Only TEs showing average RPM > 0 were considered to be targeted by sRNAs (silenced). In order to associate the obtained RPM values with all TE insertions identified in each group of populations, we first assigned an ID number to new TEs (i.e. those not included in the *A. lyrata* TE annotation file) by matching TE insertions in a range of ±50 bp. This range was decided following a previous study using a similar range to match TE coordinates (Cuenca-Guardiola et al. 2023). If no annotated TE was found in that range, we were not able to test if the TE was targeted by siRNAs or not; thus, we assigned it the label “no information.”

### Assignation of TEs to Genic Regions

We used the tool UROPA (Kondili et al. 2017) and the *A. lyrata* v2.1 genome annotation (Phytozome) in order to know which TE insertions were located nearby/inside genes, and if so, which part of the gene. The TE insertions were annotated using all genes and their features that overlapped the TE insertions and also by all genes from which the insertion was 2 kb upstream or downstream. Accordingly, the genomic locations considered were: upstream, 5′ UTR, CDS, 3′ UTR, intron, and downstream. TE insertions with no associated genes were annotated as intergenic (Table 2).

### Gene Expression Data

In order to identify endosperm- and ubiquitous-specific genes, we used RNAseq expression data from various tissues, including leaf, root, pistil, and pollen, obtained from populations of European and North American lineages of *A. lyrata* as generated by İltaş et al. (2024). Additionally, we incorporated endosperm RNAseq data from five outcrossed *A. lyrata* individuals, derived from a previous study by Klosinska et al. (2016). The endosperm data were integrated into the data processing pipeline, as described by İltaş et al. (2024). Genes were considered as endosperm-specific if they showed significant upregulation (log_2_FC > 1 and adjusted *P* < 0.05) in all pairwise comparisons with other tissues (supplementary table S9, Supplementary Material online). We did the same for all tissues. We then considered as ubiquitous the genes that did not show a tissue-specific pattern according to these criteria; to eliminate silenced genes and pseudogenes, we added another criterion for ubiquitous genes to have a minimum average of 20 read counts across all RNA-Seq libraries. The RNAseq data for leaf, root, pistil, and pollen tissues can be found in the NCBI BioProject database under submission number PRJNA1067428 (İltaş et al. 2024). The endosperm RNAseq data are available under submission number GSE76076 (Klosinska et al. 2016). Finally, for imprinted genes, we collected the list of maternally and paternally expressed genes from a previous study (Klosinska et al. 2016).

### Insertion Frequency Spectrum

For TEs under purifying selection, we expect an excess of rare insertions (Nielsen and Slatkin 2014). If there is a stronger purifying selection against silenced TEs, we expect a higher proportion of rare silenced TEs than nonsilenced TEs. If genomic imprinting is under positive selection, we expect an excess of fixed silenced TEs associated with imprinted genes compared with those associated with endosperm and ubiquitous genes. To test these assumptions, we generated the IFS of silenced and nonsilenced TEs associated with imprinted genes, endosperm, and ubiquitous genes for each group of populations of *A. lyrata* (European, core, edge, and selfer). We calculated 95% confidence intervals from 1,000 bootstraps of each TE category. All statistical analyses and permutation tests were performed in R.

## Supplementary Material

msaf093_Supplementary_Data

## Data Availability

Nanopore long-read sequencing data for the three plant individuals—RON5.9 (sample SAMN43781236), TSS14.18 (sample SAMN43781228), and INDP15.1 (sample SAMN43781226)—are available in EBIN/ENA at https://www.ebi.ac.uk/ena, and can be accessed under project PRJEB80457. The detailed information about the identified TE insertions is available in a dataset in Figshare (dx.doi.org/10.6084/m9.figshare.28816622).
