# Supplementary material for: The Demographic History of Populations and Genomic Imprinting have Shaped the Transposon Patterns in Arabidopsis lyrata
Source: Mol Biol Evol. 2025 Apr 24;42(5):msaf093. doi: 10.1093/molbev/msaf093 (PMC12159739; doi:10.1093/molbev/msaf093)
Supplement: msaf093_Supplementary_Data [file msaf093_supplementary_data.zip › Figure S8.pdf]

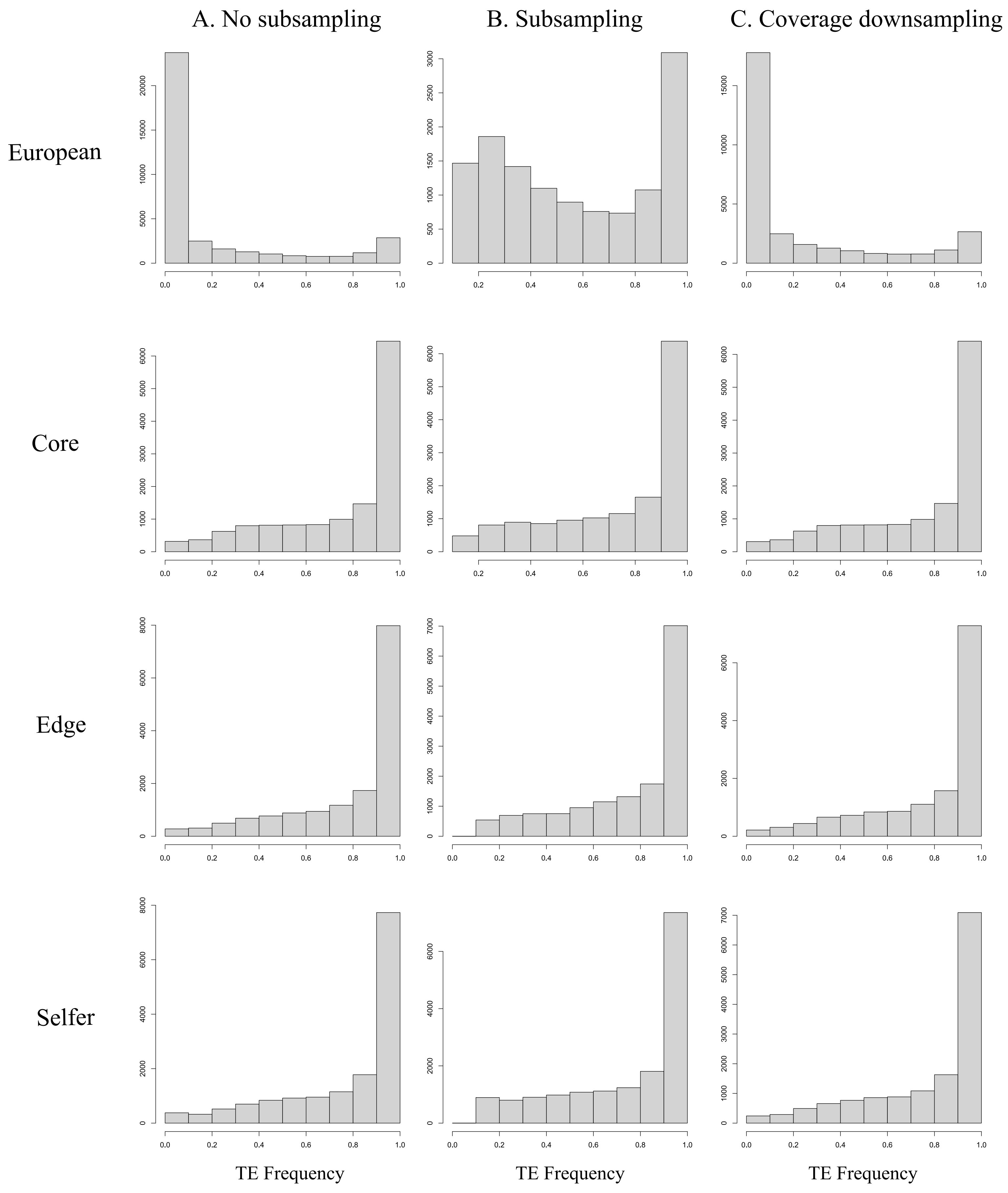
A. No subsampling
B. Subsampling
C. Coverage downsampling
European
Core
Edge
Selfer
TE Frequency
TE Frequency
TE Frequency
