# Supplementary figures and images for: The Demographic History of Populations and Genomic Imprinting have Shaped the Transposon Patterns in Arabidopsis lyrata
Source: Mol Biol Evol. 2025 Apr 24;42(5):msaf093. doi: 10.1093/molbev/msaf093 (PMC12159739; doi:10.1093/molbev/msaf093)

**A**

Percentage of TEs according to orders

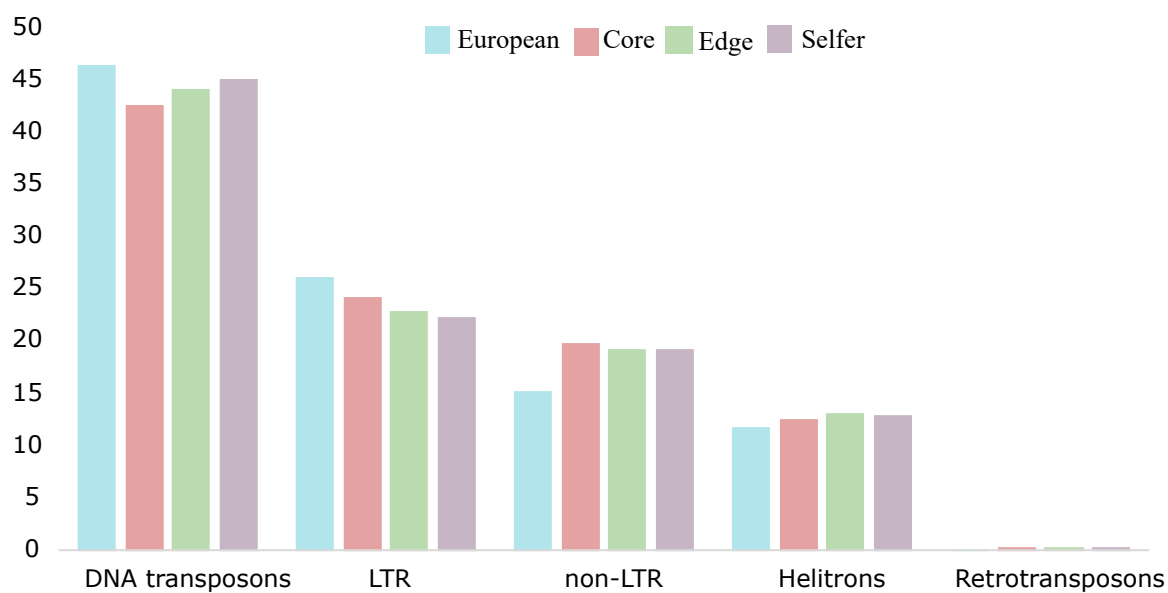**B**

Percentage of TEs according to superfamilies

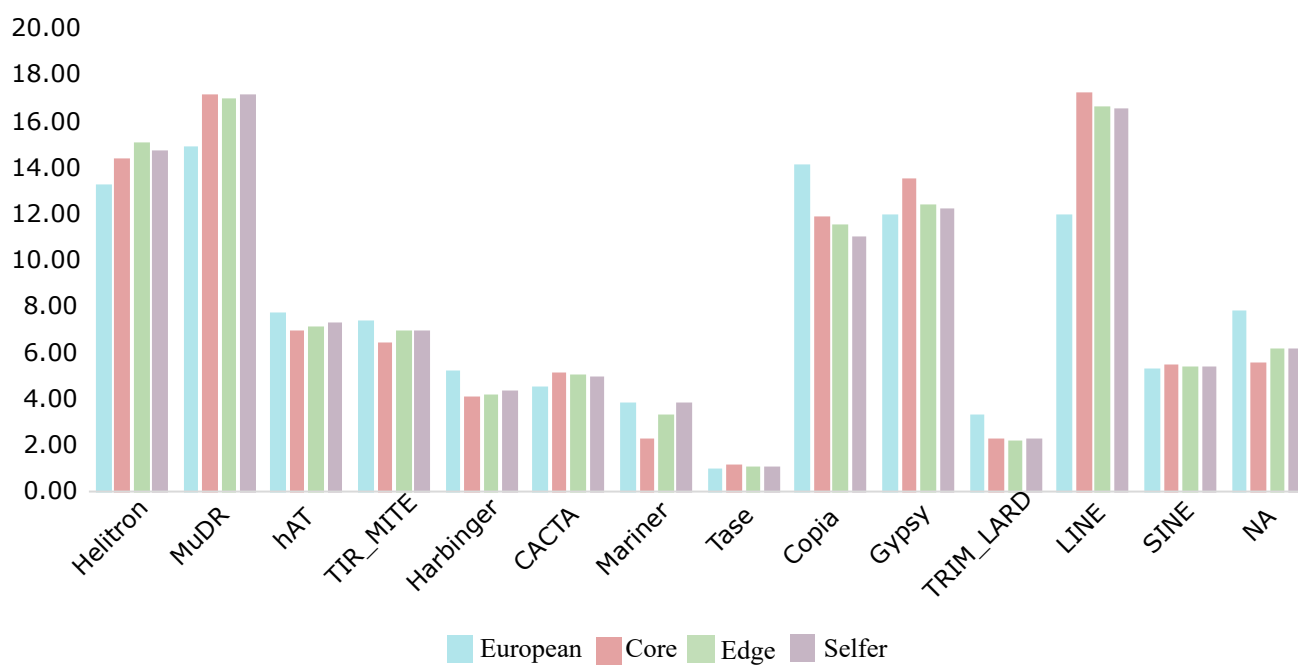

Supplement: msaf093_Supplementary_Data [file msaf093_supplementary_data.zip › Figure S1.pdf]

Distribution of TE frequency according to population group

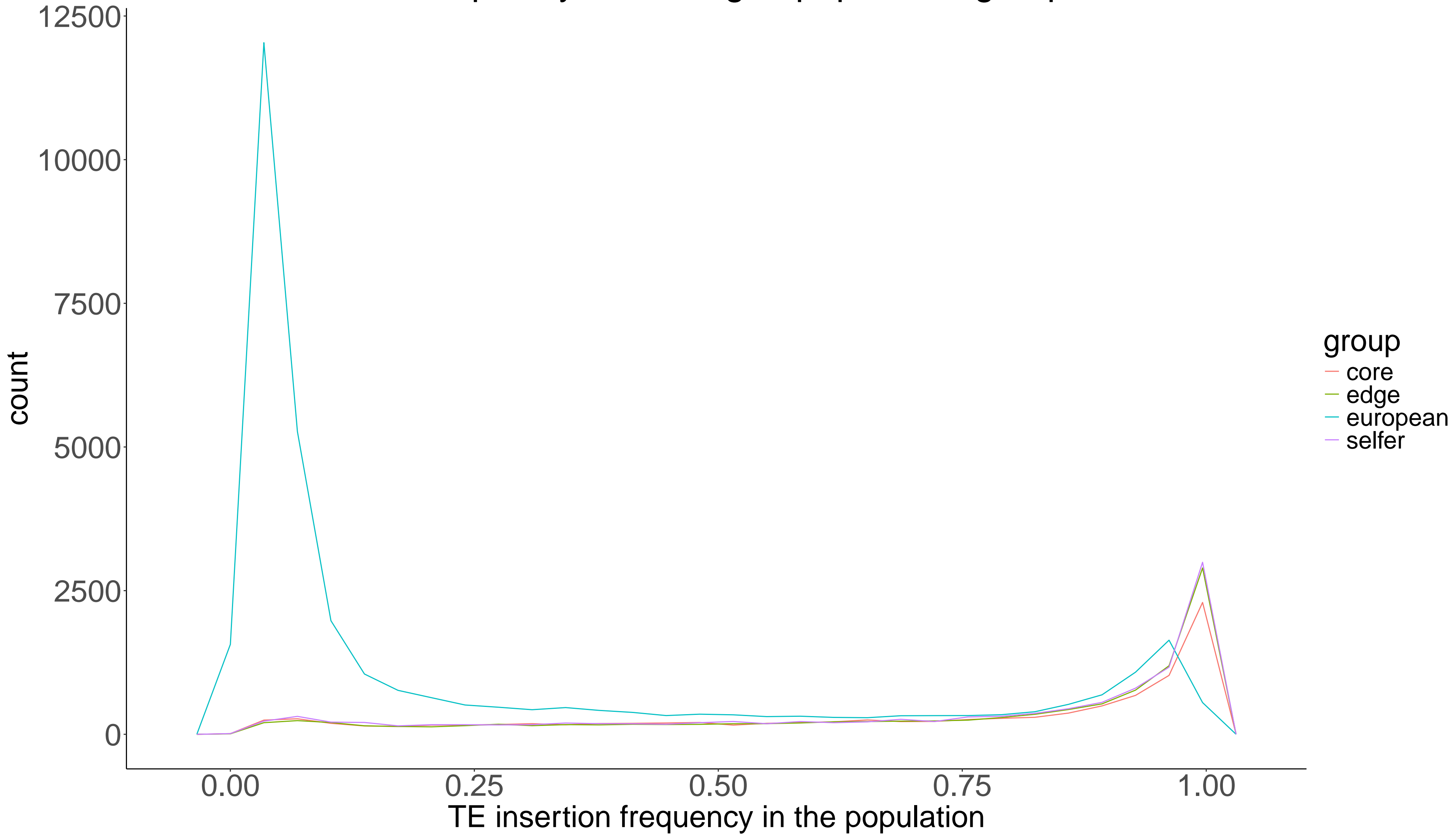

Supplement: msaf093_Supplementary_Data [file msaf093_supplementary_data.zip › Figure S2.pdf]

## A. Selfer

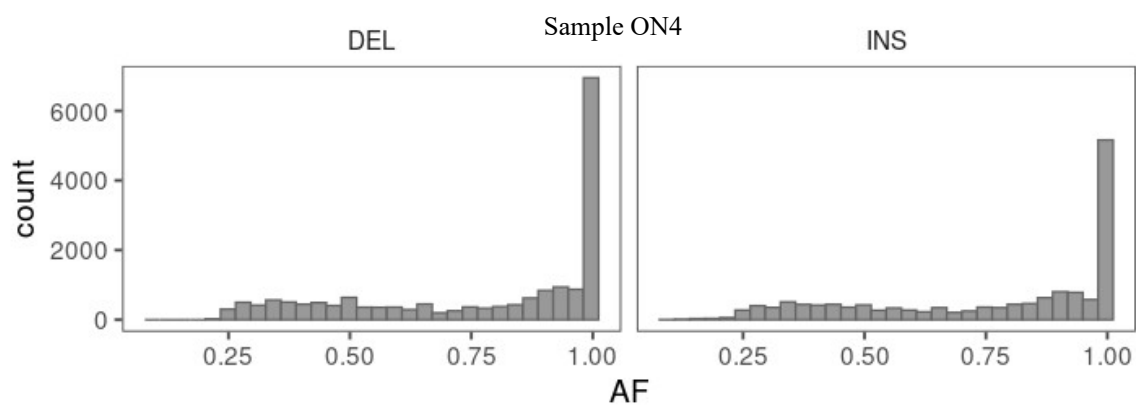

## B. Edge

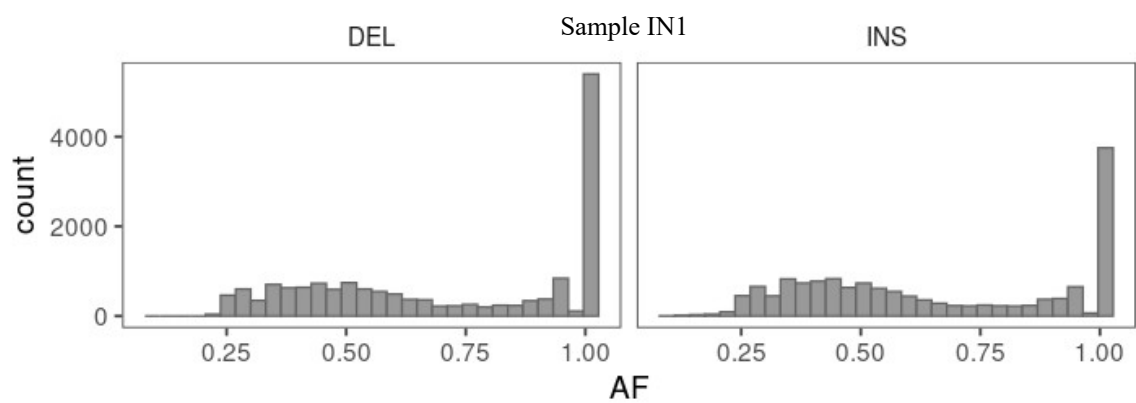

## C. Edge

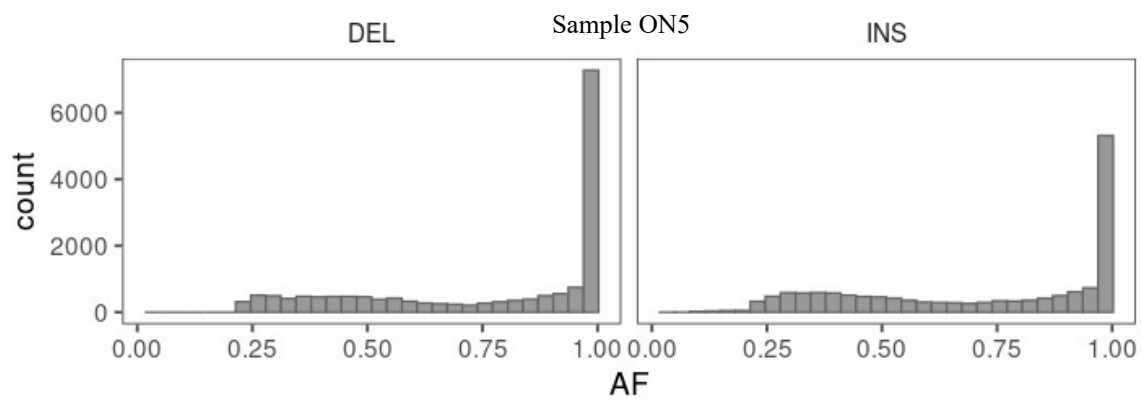

Supplement: msaf093_Supplementary_Data [file msaf093_supplementary_data.zip › Figure S3.pdf]

A. TEs - Insertions SVs

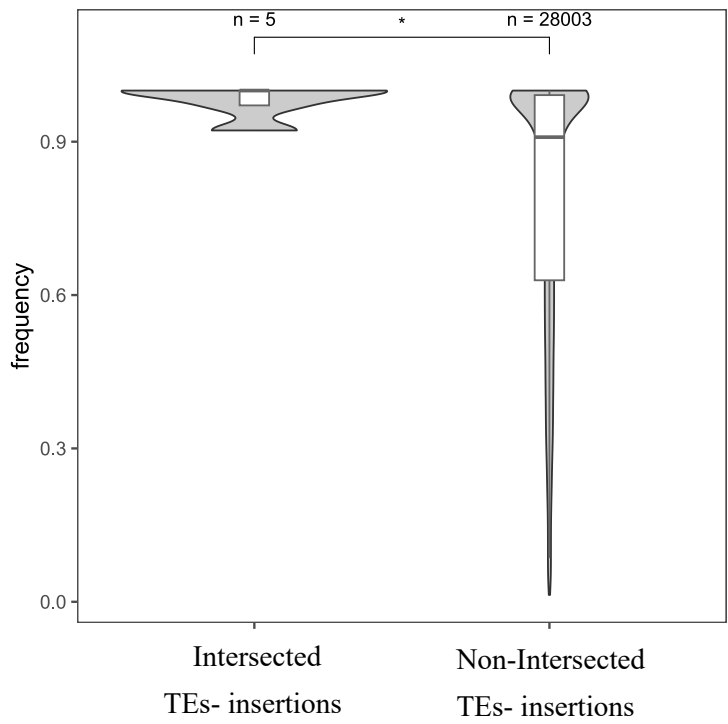

B. TEs - Deletions SVs

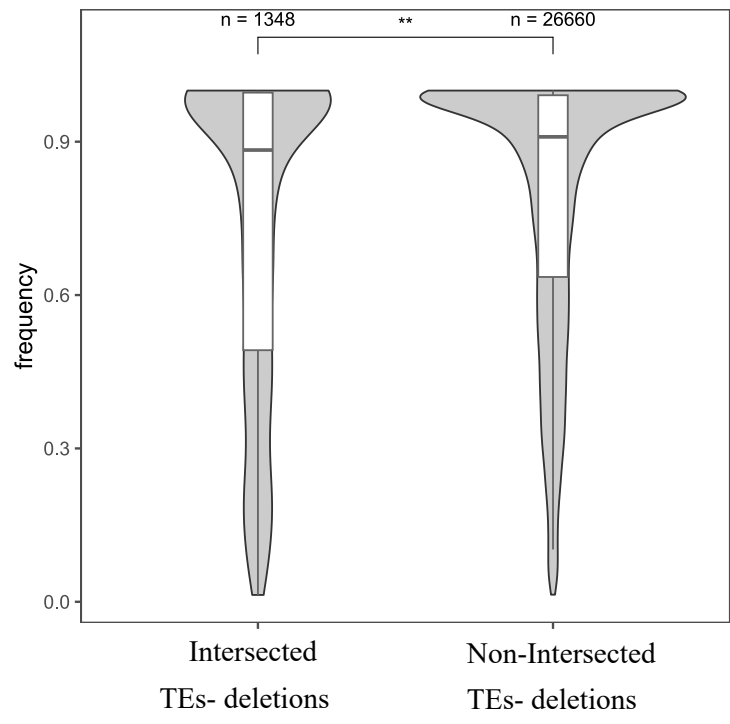

Supplement: msaf093_Supplementary_Data [file msaf093_supplementary_data.zip › Figure S4.pdf]

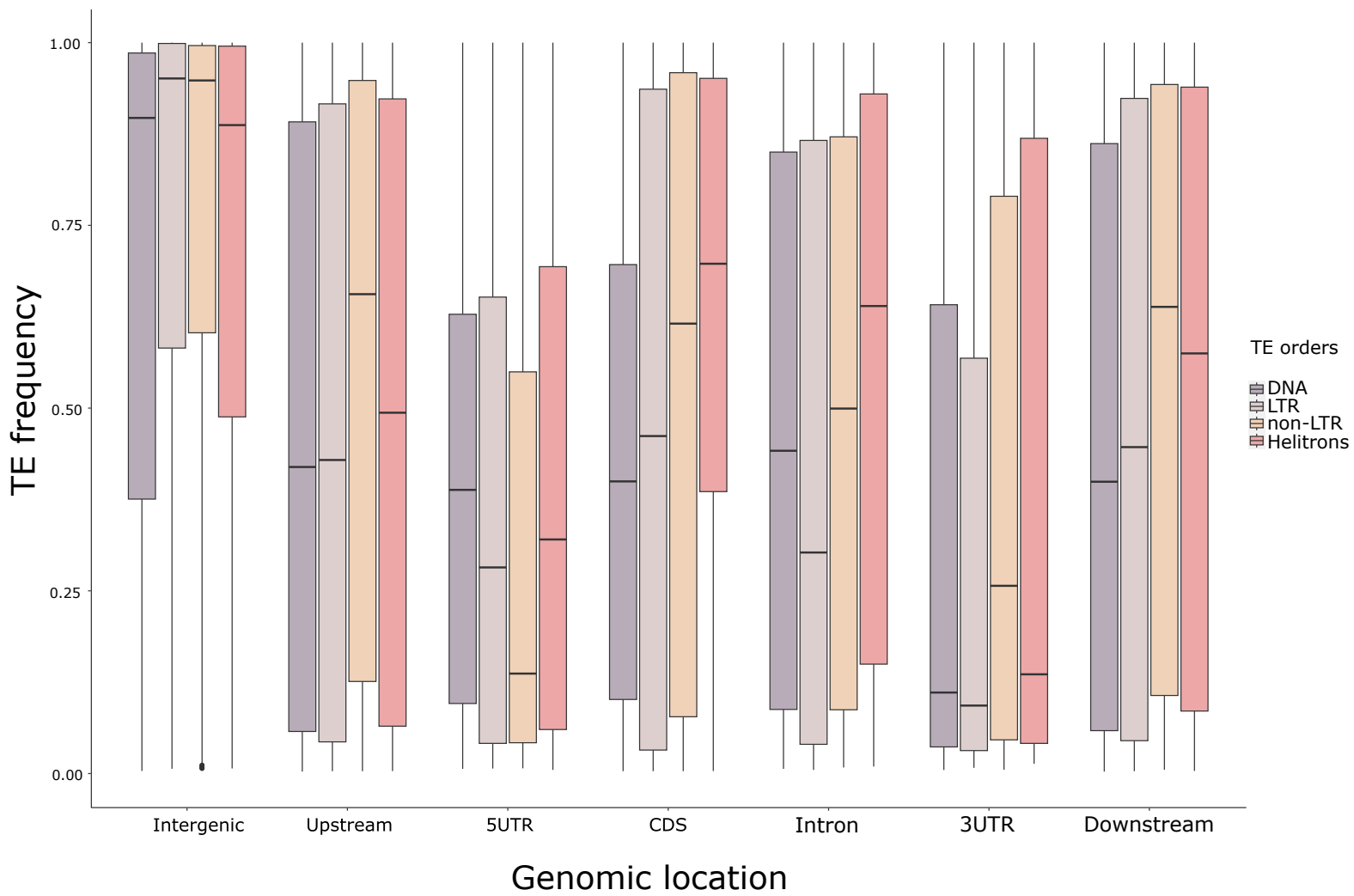

Supplement: msaf093_Supplementary_Data [file msaf093_supplementary_data.zip › Figure S5.pdf]

## A. All TEs

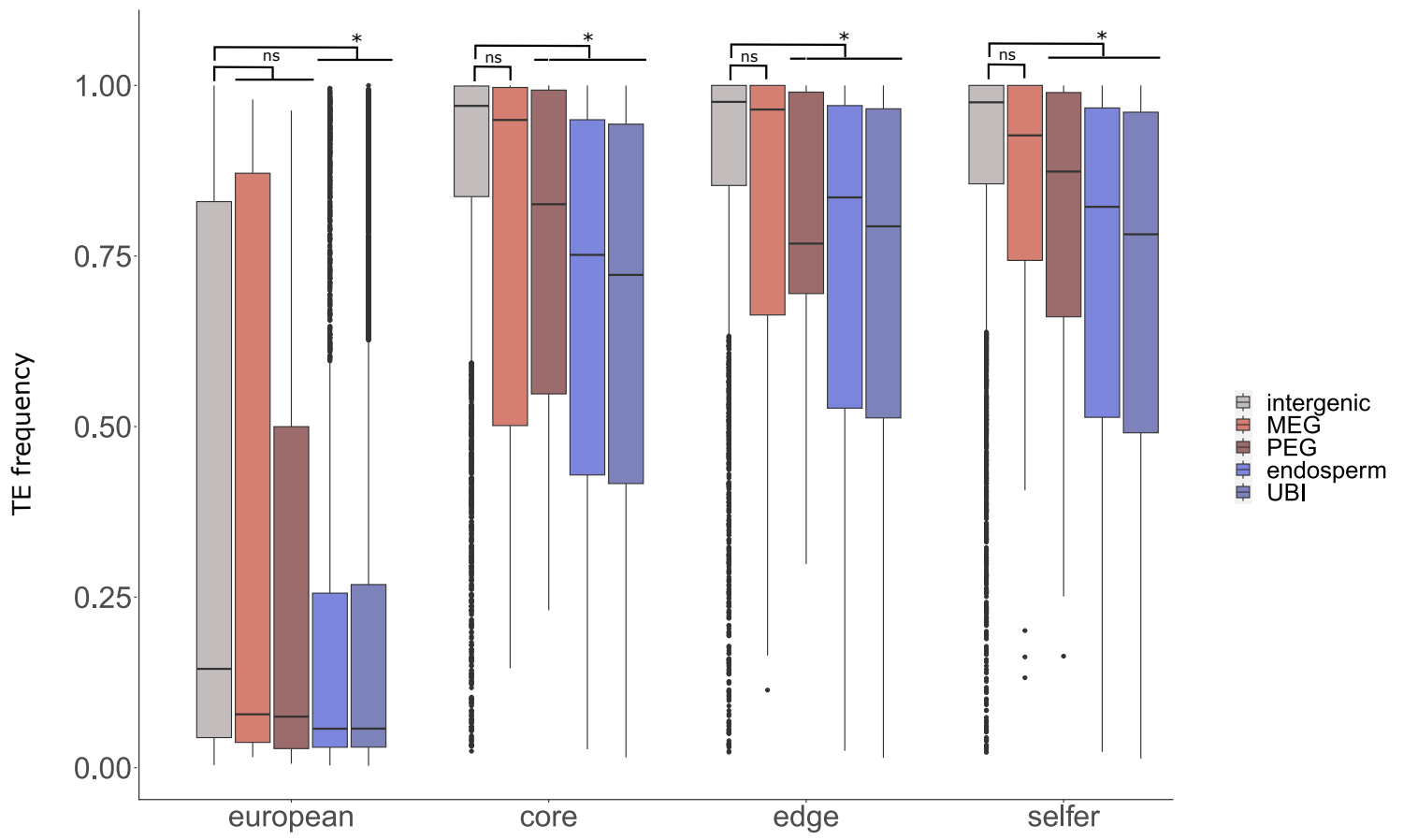

Supplement: msaf093_Supplementary_Data [file msaf093_supplementary_data.zip › Figure S6.pdf]

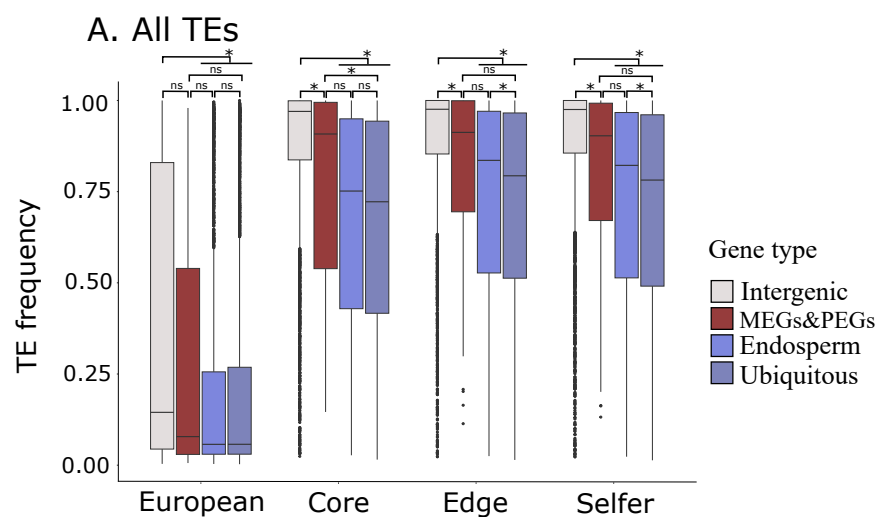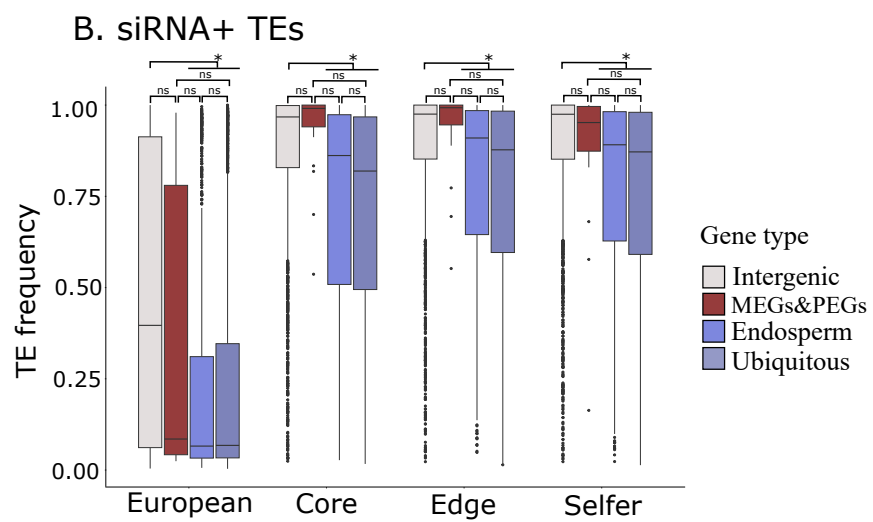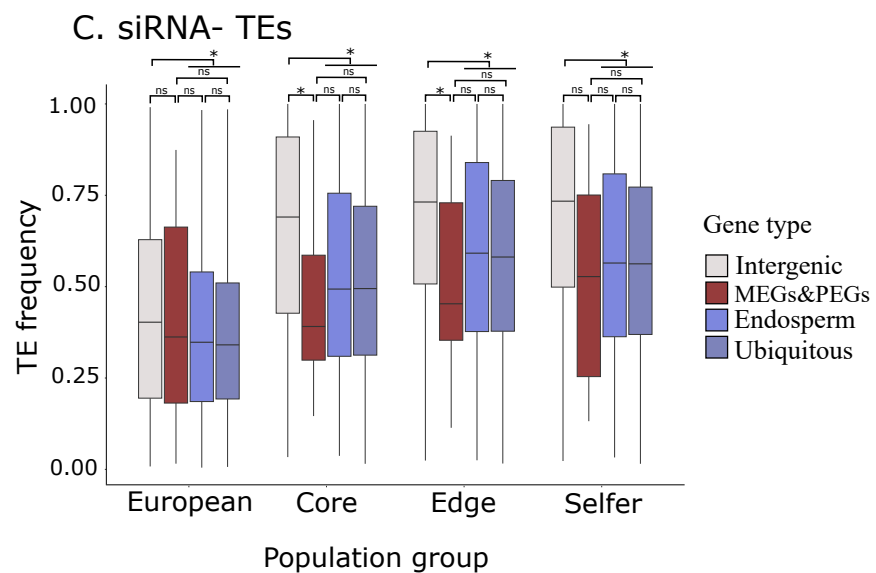

Supplement: msaf093_Supplementary_Data [file msaf093_supplementary_data.zip › Figure S7.pdf]

A. No subsampling

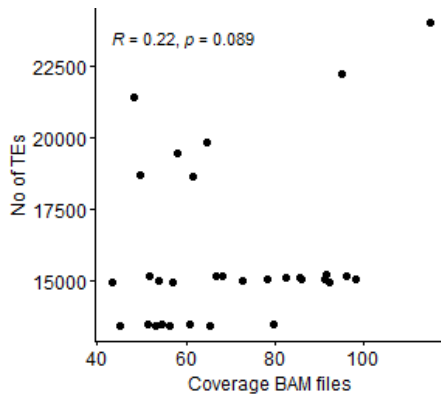

B. Subsampling ppileup

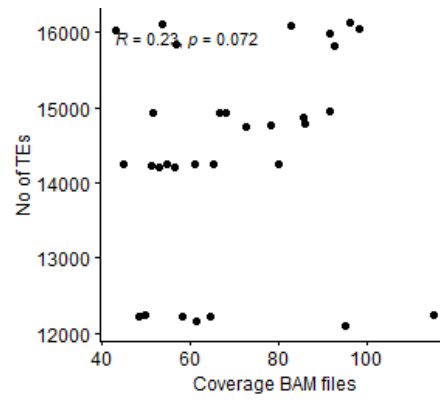

C. Downsampling BAM files coverage

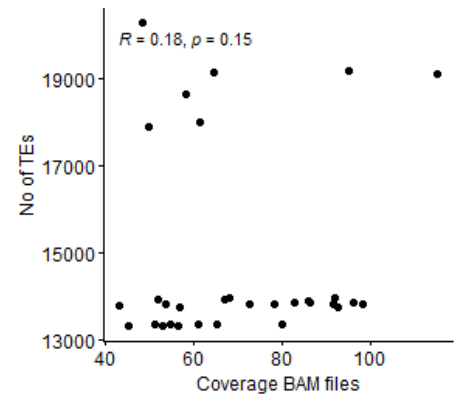

Supplement: msaf093_Supplementary_Data [file msaf093_supplementary_data.zip › Figure S9.pdf]
